# Glycogenic Hepatopathy: Resolution with Minimal Glucose Control

**DOI:** 10.1155/2017/7651387

**Published:** 2017-04-26

**Authors:** Abhimanyu Chandel, Brittany Scarpato, Jeanette Camacho, Miles McFarland, Shaffer Mok

**Affiliations:** Gastroenterology Department, Cooper University Hospital, Cooper Medical School of Rowan University, Camden, NJ, USA

## Abstract

We describe a presentation of glycogenic hepatopathy in a poorly controlled type I diabetic patient. As patients with glycogenic hepatopathy often have nonspecific complaints, diagnosis tends to be delayed and laboratory and imaging data are often indistinguishable from nonalcoholic fatty liver disease. Our patient's diagnosis of glycogenic hepatopathy required a liver biopsy, which demonstrated the characteristic pathology. Her symptoms resolved with minimal alteration to her insulin regimen and only slightly improved glucose control.

## 1. Introduction

Diabetes is associated with several etiologies of hepatic dysfunction. Type 1 diabetes mellitus (T1DM) is more commonly associated with glycogenic hepatopathy (GH) and T2DM with nonalcoholic fatty liver disease (NAFLD) [1]. These disease processes are not easily distinguished based on patient presentation or ultrasound and differentiation often requires a liver biopsy [1].

However, prior to biopsy, viral, autoimmune, and underlying metabolic liver disease should be excluded via corresponding laboratory investigations. If such processes are excluded, differentiation between NAFLD and glycogenic hepatopathy is essential to guide appropriate management, as NAFLD can progress to advanced liver disease [1–6].

## 2. Case Report

A 12-year-old female with a history of poorly controlled T1DM (most recent HgbA1c of 10.5%), frequent hospitalizations for diabetic ketoacidosis, and psoriasis presented to her endocrinologist for routine checkup. One week ago, she experienced a 30-minute episode of sharp upper abdominal pain. The patient denied trauma, alcohol, acetaminophen use, or previous blood transfusions. Both her parents were immunized for hepatitis and there was no family history of liver, gastrointestinal, or autoimmune diseases.

Her physical exam and symptoms of sharp abdominal pain prompted further evaluation. Liver function tests revealed AST 690 U/L, ALT 356 U/L, total bilirubin 0.2 mg/dL, and ALP 158 U/L. Autoimmune tests, viral hepatitis panel, and acetaminophen level were within normal limits. MRI revealed a liver span of 20.7 cm craniocaudally with marked hypertrophy of the left hepatic lobe. Given the patient's personal history of autoimmune disease, autoimmune hepatitis was considered high in the differential and a liver biopsy was performed.

**Figure 1:**
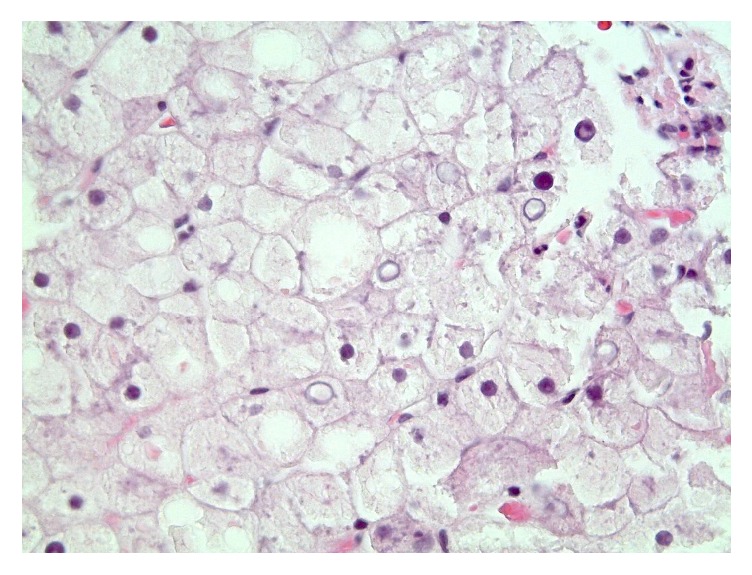
Liver biopsy specimen. Portal triad in center. Hepatocytes have pale faintly granular eosinophilic to clear cytoplasm. Note that some nuclei have cleared chromatin (nuclear glycogenosis), which can be seen in various metabolic conditions, such as diabetes mellitus (Hematoxylin and Eosin staining at 400x magnification).

**Figure 2:**
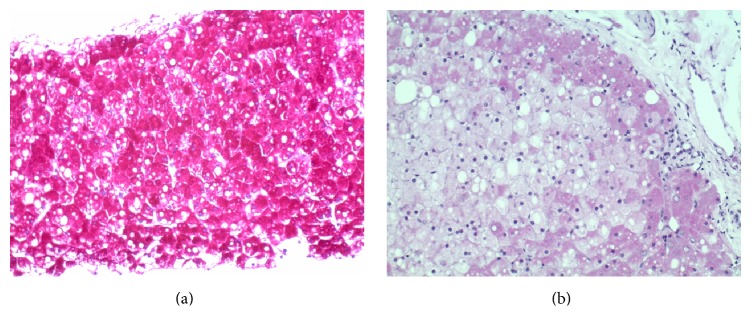
(a) 200x magnification PAS stain (without diastase) to show bright red staining of hepatocyte cytoplasm. (b) 200x PAS stain with diastase (to break down glycogen) to show marked decrease in bright red staining of hepatocyte cytoplasm, indicating the amount of glycogen present before. The clear vacuoles are consistent with lipid (steatosis).

Microscopically, the biopsy demonstrated pale hepatocytes with diffuse hepatocyte ballooning. Nuclear glycogenosis was observed (Figure 1). Periodic acid-Schiff stain revealed glycogen accumulation which dissolved upon diastase application (Figure 2). Reticulin stain demonstrated compressed sinusoids. Mild steatosis, mostly macrovesicular, was present. No inflammatory infiltrates or fibrosis by reticulin or trichrome stain was demonstrated. Iron stain was negative. These findings, in conjunction with coexisting poorly controlled T1DM, were consistent with glycogenic hepatopathy.

As the origin of the problem was attributed to her poorly controlled diabetes, the patient's insulin regimen was carefully examined and a more aggressive correction formula was added to the patient's basal insulin. Three months later, her HgbA1c remained significantly elevated at 10.3%. Despite this minimal improvement in glucose control, AST and ALT dropped to 169 U/L and 139 U/L, respectively, and no further symptoms were observed. Unfortunately, our patient never returned for additional follow-up.

## 3. Discussion

In addition to T1DM, other etiologies of GH have been described. The use of high-dose corticosteroids may precipitate or exacerbate GH. Additionally, patients with T2DM requiring insulin replacement therapy may also develop GH. A case has also been reported in a patient following intentional insulin overdose [6, 7].

### 3.1. Pathogenesis

In the setting of poorly controlled T1DM, the presence of excess glucose and insulin contributes to glycogen storage in the liver. Insulin activates glycogen synthase phosphatase, which is responsible for dephosphorylating and activating glycogen synthase. Glycogen synthase converts glucose 1-phosphate into glycogen and increases glycogen storage in the liver while inhibiting glycogenolysis [1, 2, 6]. Additionally, frequent hypoglycemic episodes may exacerbate GH, as these episodes are treated with glucose administration and may promote further glycogen formation and deposition [6].

A patient with GH may present with hepatomegaly, abdominal pain, nausea, vomiting, and abnormal liver function tests reflecting acute liver injury [1–9]. The extent of transaminase elevation varies; cases have been reported with sudden elevation of transaminases to 30 times the upper normal range, mimicking acute viral hepatitis [8]. The etiology for hepatomegaly is less clear and may be a complication of diabetes [3].

### 3.2. Imaging

Case reports have not demonstrated success in differentiating glycogenic hepatopathy from NAFLD with ultrasound or other imaging modalities. Differentiation is further complicated by possible coexistence of steatosis with glycogenic hepatopathy [1, 2]. Overall, in these series, an ultrasound was unable to distinguish glycogen accumulation from fat deposition [7].

Sweetser and Kraichely discussed diagnostic clues provided by CT scan to help differentiate NAFLD and GH [9]. In this report, imaging of NAFLD demonstrated a hypodense liver on CT scan, whereas GH presented with a hyperdense liver [9]. However, other possible etiologies for increasing hepatic attenuation on unenhanced CT include conditions where radiodense material is deposited in the liver, such as iodine (in patients taking amiodarone) and iron in patients with hemochromatosis [9]. Additionally, a hyperdense liver on CT scan may not be sufficiently sensitive for diagnosis. Glycogen deposition may not be uniform throughout the liver and concomitant hepatic processes such as shock liver or acute liver injury often present with hypodense liver on CT scan, complicating the diagnosis of GH via imaging [7].

### 3.3. Pathology

Histology of GH reveals pale appearance of hepatocytes with compression of sinusoids, glycogenated nuclei, giant mitochondria, and positive periodic acid-Schiff stained intracytoplasmic inclusions that disappear after digestion with diastase [1–3, 6].

Conversely, nonalcoholic steatohepatitis demonstrates a degree of steatosis with lobular inflammation, portal inflammation, hepatocyte ballooning, and/or fibrosis [6].

### 3.4. Management and Prognosis

Patients presenting with tender hepatomegaly and abnormal liver function tests without other identifiable causes should undergo early liver biopsy. It may be just as crucial to consider biopsy in poorly controlled diabetic patients with mildly elevated liver enzymes to avoid misdiagnosis and the ultimate misutilization of medical resources if the diagnosis of NAFLD is made erroneously on a solely clinical basis [6, 8]. In some patients with poorly controlled diabetes and mild abnormalities in liver function tests, it is reasonable to defer biopsy until following a trial of improved glycemic control. However, signs of recurrent liver damage after short term improvement in glycemic control have been noted and should prompt consideration of biopsy to guide further management [2].

Once the diagnosis has been confirmed, improved glycemic control is the mainstay of management. Unlike hepatic steatosis that progresses to fibrosis, GH demonstrates reversibility, which occurs, almost uniformly, with improved glycemic control [1, 2, 5, 6, 8]. Improvement has been demonstrated within 4 weeks of optimal glycemic control using insulin treatment in patients with GH [1]. Continuous subcutaneous insulin infusion has been successfully used to resolve glycogenic hepatopathy in one case [4]. Furthermore, persistent reversal has been observed following pancreas transplantation [2].

Prognosis with improved glycemic control is excellent. Though management is aimed at better glycemic control, the degree of improvement needed is unclear. Cha et al. presented three cases with improvement of GH and resolution of transaminitis without aggressive insulin treatment. Patients in this series had resolution of GH without HgbA1c decreasing below 11% [8]. In some cases, though symptoms and histopathology may resolve with improved glycemic control, transaminitis may persist [6]. Parmar et al. demonstrated that a drop of 0.6% in HgbA1c (from 13.3% to 12.7%) provided symptomatic relief for their patient with GH [6]. Persistently elevated liver enzymes following improved glycemic control should prompt evaluation for an additional or alternative disorder.

This case illustrates GH in a type I diabetic diagnosed via the characteristic pathology and treated successfully despite only modest improvements in glucose control. Unfortunately, further analysis of our patient's case and confirmation of resolution of transaminitis are limited by incomplete follow-up. Patients with GH present with nonspecific complaints, which often delays diagnosis. As laboratory and imaging studies often do not successfully differentiate GH from NAFLD, a biopsy may be required. It is important to consider GH when caring for T1DM patients presenting with elevated transaminases as the natural history and management differ from that of NAFLD.

## Additional Points

Case was treated and written while all authors were affiliated with Cooper University Hospital in Camden, New Jersey.
